# Role of the Epipapillary Membrane in Maculopathy Associated with Cavitary Optic Disc Anomalies: Morphology, Surgical Outcomes, and Histopathology

**DOI:** 10.1155/2018/5680503

**Published:** 2018-05-08

**Authors:** Atsushi Tanaka, Wataru Saito, Satoru Kase, Kan Ishijima, Kousuke Noda, Susumu Ishida

**Affiliations:** ^1^Department of Ophthalmology, Faculty of Medicine and Graduate School of Medicine, Hokkaido University, Sapporo, Japan; ^2^Enoki Eye Clinic, Sayama, Japan; ^3^Kaimeido Eye and Dental Clinic, Sapporo, Japan

## Abstract

**Purpose:**

To evaluate the surgical outcomes of pars plana vitrectomy (PPV) with epipapillary membrane removal in patients with maculopathy associated with cavitary optic disc anomalies.

**Methods:**

Eight patients (8 eyes) with cavitary optic disc anomaly-associated maculopathy who underwent PPV with epipapillary membrane removal were retrospectively reviewed. The best-corrected visual acuity (BCVA) and macular and papillary morphologies using enhanced depth imaging optical coherence tomography (EDI-OCT) were evaluated before and after treatment. Immunohistochemistry for an intraoperatively excised epipapillary membrane tissue was also performed.

**Results:**

Before surgery, EDI-OCT revealed that epipapillary membrane was observed in all patients. Retinoschisis was resolved with no recurrence in all patients following vitrectomy regardless of a disease type or the presence or absence of preoperative posterior vitreous detachment. The mean final BCVA and central retinal thickness significantly improved compared with pretreatment values (*P* = 0.008 and 0.004, resp.). Immunoreactivity for S100 protein and glial fibrillary acidic protein, markers of astrocytes, was positive in the resected membrane tissues.

**Conclusions:**

These results suggest that epipapillary membrane is involved in the pathogenesis of some patients with cavitary optic disc anomaly-associated maculopathy as well as posterior hyaloid membrane. PPV with epipapillary membrane removal may be a useful treatment option for this maculopathy. This trial is registered with UMIN000011123.

## 1. Introduction

Optic disc pit (ODP) and morning glory disc disorders are congenital cavitary anomalies of the optic disc, which are associated with dysraphism of the optic fissure. Disc anomalies have common histopathologic characteristics: defects of the lamina cribrosa and the sclera, invagination of the retinal tissue into the excavation of the optic disc, attachment of the vitreous cortex and glial tissue to the invaginated retinal tissues, and presence of the subarachnoid space directly under the excavation site of the disc [1–3]. These disc anomalies can also involve retinal schisis extending from the optic disc to the posterior pole, with often macular serous retinal detachment (SRD) [1, 3, 4]. However, the etiology that causes retinal schisis in these disc anomalies is not fully elucidated.

The posterior hyaloid membrane and the vitreous strand, which is possibly a remnant of the Cloquet's canal, are attached to the site with the pit [5–8]. In patients with ODP maculopathy without preoperative posterior vitreous detachment (PVD), indeed, retinal schisis is resolved in most cases after pars plana vitrectomy (PPV) with the generation of PVD, but without any internal limiting membrane peeling, retinal photocoagulation at the disc edge, or gas tamponade [9, 10]. Retinal schisis disappeared after spontaneous development of PVD [11]. These observations suggest that a certain communication between the retinal schisis and the vitreous cavity or subarachnoid space arose by which the posterior hyaloid membrane drew the vulnerable disc tissue [1, 12]. However, there were cases with no improvement or showing recurrence of the retinal schisis, even after PVD was made following PPV [9, 13, 14]. In case series with a long-term follow-up, a foveal reattachment rate after PPV with induction of PVD was 81% and internal limiting membrane (ILM) peeling was not associated with foveal reattachment [14]. Furthermore, the retinal schisis did not improve despite performing PPV in all cases with preoperative PVD [15]. A recent study reported surgical outcomes of PPV with creation of inner retinal fenestration [16]. Retinal schisis is resolved after PPV in almost all cases, even though both induction of PVD and ILM removal were not performed [16]. However, multicentered studies examining long-term outcomes of this new surgical procedure are needed. Thus, these observations suggest that there is another unidentified pathological mechanism other than the traction of the hyaloid membrane.

A recent study using spectral domain optical coherence tomography (SD-OCT) demonstrated that all eyes with ODP involved epipapillary membrane, which developed over time [17]. Although the membrane may be related to the pathogenesis of ODP maculopathy by dragging the disc, surgical outcomes of PPV with epipapillary membrane removal have previously been reported in only one case [18].

In patients with morning glory syndrome, a study using SD-OCT reported the detection of attached posterior hyaloid membrane and glial tissues at the excavation site of the disc and a good surgical outcome following PPV after the removal of these tissues [4]. This study suggests that the traction of the hyaloid membrane and/or glial tissues is involved in the pathogenesis of the morning glory syndrome.

Glaucomatous optic disc is an acquired cavitary anomaly of the optic disc. Though rarely, eyes with glaucoma involve retinal schisis, despite the absence of the pit within the disc [19–22]. Retinal schisis disappeared or improved after PPV with the generation of PVD in patients with retinoschisis associated with glaucoma without preoperative PVD [22]. Furthermore, retinal schisis was resolved following PPV with the removal of the glial tissue present on the disc in a patient with preoperative PVD [21]. These studies suggest that the posterior hyaloid membrane and/or glial tissue on the disc is related to the pathogenesis of retinoschisis associated with glaucoma. Taken together, previous findings suggest that traction force to the fragile optic disc of the posterior hyaloid membrane and/or epipapillary membrane is involved in the pathogenesis of maculopathy associated with congenital or acquired optic disc anomalies. Therefore, we hypothesized that PPV that removes not only the hyaloid membrane but also the epipapillary membrane would be effective for treating maculopathy associated with cavitary optic disc anomalies [23]. The purpose of this study was to evaluate the surgical outcomes of PPV with epipapillary membrane removal and to analyze histopathology of the resected membrane in order to examine the role of epipapillary membrane in patients with maculopathy associated with cavitary optic disc anomalies.

## 2. Methods

### 2.1. Patients

We retrospectively reviewed medical records of eight successive cavitary optic disc anomaly-associated maculopathy patients (eight eyes) that could be followed up for more than 6 months after PPV with epipapillary membrane removal. In addition to patients with maculopathy associated with congenital cavitary optic disc anomalies, we included patients with maculopathy associated with glaucoma as subjects of this study due to the following reasons: (1) this maculopathy has similar features with ODP maculopathy in anatomical vulnerability at the optic disc [1, 2, 24] and the area of retinal schisis; (2) we encountered successful postsurgical results in a patient with retinoschisis associated with glaucoma that underwent PPV with epipapillary membrane removal [21]. During the period extending from June 2009 to March 2015, we encountered nine patients with this syndrome at the vitreoretinal clinic of Hokkaido University Hospital. One case that was followed up for less than 6 months after surgery was excluded from this study. The current study was approved by the Ethics Committee of Hokkaido University Hospital (013-0098) and followed the tenets of the Declaration of Helsinki. Written informed consent was obtained from all patients after an explanation of the purpose and procedures of this study.

### 2.2. Surgical Procedures

All patients underwent standard three-port transconjunctival PPV using a 23- or 25-gauge trocar system. One surgeon (W.S.) performed PPV in all patients. The surgical procedures included the intentional generation of PVD except for patients with preoperative PVD, followed by vitreous gel excision to the equator using triamcinolone acetonide. In all cases, the ILM was peeled off in a range from the macular area to the margin of the optic disc, after being stained with Brilliant Blue G. Thereafter, the presence of the epipapillary membrane tissue was ascertained using triamcinolone acetonide and was carefully removed with the forceps under a high-magnification direct contact lens. Laser photocoagulation for the surrounding retina of the optic disc or intravitreal sulfur hexafluoride (SF_6_) gas injection for macular retinal schisis/retinal detachment was not planned in all cases. Cataract surgery was concurrently performed at the time of PPV in six eyes. No patients suffered postoperative complications that could potentially affect OCT imaging during follow-up, such as hazy cornea, dislocated intraocular lens, or posterior capsule opacity.

### 2.3. Ophthalmic Examinations

On the initial visit, all patients underwent a complete ophthalmic examination including decimal best-corrected visual acuity (BCVA) with a Japanese standard Landolt visual acuity chart, color fundus photography, SD- and enhanced depth imaging (EDI-) OCT (RS-3000 and RS-3000 Advance with software version NAVIS-EX 1.3.7; NIDEK, Gamagori, Japan), and single-flash electroretinography (ERG). Fluorescein angiography was performed in all the eyes except for case 1. Central retinal thickness (CRT) using OCT was determined by manually measuring the distance between the inner limiting membrane and the retinal pigment epithelium at the fovea. During follow-up, BCVA, SD-OCT, and EDI-OCT were performed at every visit. The changes in BCVA, morphology of the retina and optic disc on SD-OCT, and CRT were evaluated before and after PPV. Humphrey threshold 30-2 perimetry was performed for three patients with glaucoma pre- and postoperatively. We defined the presence of epipapillary membrane, when all of the following 3 requirements were met: (1) the presence of hyperreflective lesion lying on the optic disc, on preoperative OCT; (2) a material attached on the disc that the operator could visually recognize and remove during surgery; and (3) optic disc cupping on postoperative OCT that became deeper than that on preoperative OCT.

### 2.4. Immunohistochemistry

Membrane tissues on the optic disc excised during vitrectomy in cases 4 and 5 were fixed with 4% paraformaldehyde in the operating room. Formalin-fixed, paraffin-embedded tissue sections with a thickness of 5 *μ*m were prepared. The slides were dewaxed, rehydrated, and rinsed in phosphate-buffered saline (PBS) twice for 10 min. Slides were submitted for hematoxylin-eosin staining and immunohistochemistry. As a pretreatment, microwave-based antigen retrieval was performed in 10 mM citrate buffer (pH 6.0). These slides were incubated with 3% hydrogen peroxide for 10 min and then with normal goat serum for 30 min. Sections were incubated with antiepiretinal membrane antigen (EMA) (monoclonal; dilution, 1 : 50; Dako, Japan), S100 (polyclonal; dilution, 1 : 50; Dako, Japan), and glial fibrillary acidic protein (GFAP) (monoclonal; dilution, 1 : 50; Dako, Japan) antibodies at 4°C overnight. Positive signals were visualized using diaminobendizine as a substrate. Slides were examined using a Keyence BZ-9000 (Keyence, Osaka, Japan) microscope.

### 2.5. Statistical Analysis

The BCVA was converted to the logarithm of the minimal angle of resolution (logMAR) scale for statistical analysis. Wilcoxon signed-rank test was used to compare mean values of logMAR BCVA and CRT before and after PPV. In all tests, *P* < 0.05 was considered significant.

## 3. Results

### 3.1. Patient Demographics

Table 1 summarizes the clinical features of eight patients with maculopathy associated with cavitary optic disc anomalies examined in the present study. Optic disc anomalies included ODP in three patients, glaucoma in three patients, morning glory anomaly in one patient, and coloboma of the optic disc in one patient. The male-to-female patient ratio was 3 : 1. All patients had unilateral pathology. The mean age at the initial visit was 62.5 ± 19.7 years (range: 20–82 years). The mean duration of clinical follow-up was 25.5 ± 14.2 months (range: 10–57 months). Systemic medical history revealed that two patients had well-controlled diabetes mellitus, one patient had systemic hypertension, and one patient had a breast cancer operation. One patient had already received topical latanoprost (case 6). Two patients were diagnosed with primary normal tension glaucoma on the initial visit, and treatment with topical travoprost was then initiated (cases 7 and 8). No patient had a relevant family history. Clinical symptoms were blurred vision at the central area in seven patients and anorthopia in one patient.

### 3.2. Ophthalmic Findings

**Figure 1:**
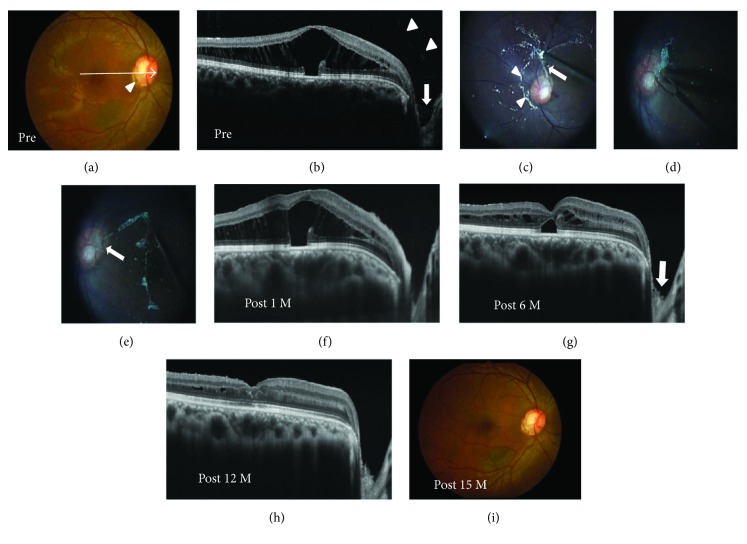
Photographs of the right eye in a 20-year-old optic disc pit (ODP) maculopathy patient without posterior vitreous detachment (PVD) (case 1). (a, b) Findings before surgery. Fundus photograph showed an ODP (a, arrowhead) and retinal elevation at the macular area (a). An arrow indicates an enhanced depth imaging optical coherence tomography (EDI-OCT) scan for the images shown in Figure 1. An EDI-OCT image revealed retinal schisis extending from the optic disc to the macula with foveal retinal detachment (b). The posterior hyaloid membrane (b, arrowheads) was attached toward the membrane that was present on the ODP (b, arrow). (c–e) Findings during pars plana vitrectomy with epipapillary membrane removal. When PVD was intentionally made, most of the hyaloid membrane was detached from the optic disc (c, arrowheads). However, a part of the membrane connected with the glial tissue on the disc (c, arrow). After making a complete PVD, the membrane tissue that remained on the ODP was grasped using forceps (d). The glial tissue was firmly attached, penetrating the ODP (e, arrow). (f–i) Postoperative OCT images. The retinal schisis gradually was reduced (f, g) and was resolved with foveal attachment 12 months after the surgery (h). The membrane tissue on the disc was also removed (g, arrow). Fundus photograph at 15 months after surgery shows the resolution of the retinal schisis (i).

**Figure 2:**
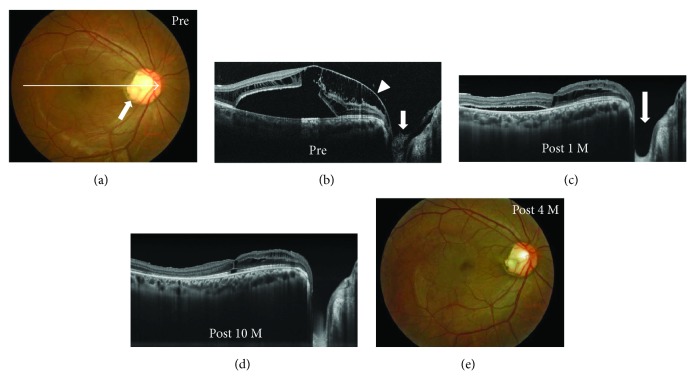
Photographs of the right eye in a 24-year-old optic disc pit (ODP) maculopathy patient without posterior vitreous detachment (case 2). (a) Fundus photograph before surgery showed an ODP (arrow) and macular retinal elevation. An arrow indicates enhanced depth imaging optical coherence tomography (EDI-OCT) scans for the images shown in Figure 2. (b–d) EDI-OCT images. Before surgery, retinal schisis extended from the optic disc to the macula with macular detachment (b). Glial tissue suggests that the Cloquet's canal (b, arrow) was also present, invaginating into the ODP and the hyaloid membrane attached to the glial tissue (b, arrowhead). The retinal schisis was reduced 1 month after pars plana vitrectomy (c) and was almost resolved with foveal attachment 10 months after surgery (d). The epipapillary membrane tissue was also removed (c, arrow). (e) The retinal elevation was reduced at four months after surgery.

**Figure 3:**
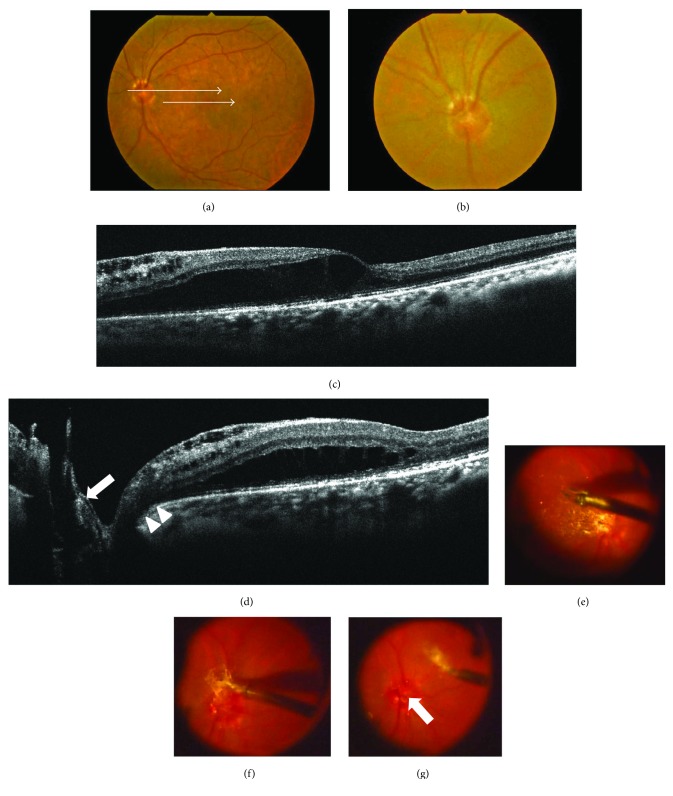
Photographs of the left eye in a 70-year-old patient with maculopathy in whom an optic disc coloboma became clear after surgery with epipapillary membrane removal (case 4). (a, b) Fundus photographs before surgery showing macular elevation (a) and a shallow cavitation in the center of the optic disc (b). Upper and lower arrows indicate the spectral domain optical coherence tomography (SD-OCT) scan in Figures 3(c) and 3(d), respectively (a). (c, d) SD-OCT images before surgery showing retinal schisis extending from the optic disc to the macula (c), with membrane tissue but with no obvious pit-like deep cavitation on the disc (d, arrow), and a shallow tunnel-like hyporeflective lesion directly connecting the retinal schisis to the disc (d, arrowheads). (e–g) Findings during pars plana vitrectomy. Internal limiting membrane (ILM) at the area from the macula to the surrounding of the disc was peeled (e). ILM connected the membrane tissue on the optic disc (f). When the epipapillary membrane tissue was removed, a pit-like concavity, which was hidden behind the tissue, appeared (g, arrow).

The mean preoperative refractive error was −0.03 ± 0.63 diopter. Slit-lamp examination showed no abnormal findings in the anterior segment of all patients and incipient cataract in five patients. The average intraocular pressure was 15.7 ± 4.0 mmHg (range: 11.3–21.0 mmHg) at the initial visit. Funduscopic examination revealed retinal elevations extending from the optic disc to the macular area in the affected eyes of all patients (Figures 1(a)–3(a), 5(a), and 6(a)). An optic disc coloboma without maculopathy (case 4) and morning glory disc anomaly with macular degeneration, suggesting the presence of a previous morning glory syndrome (case 5), were observed in each of the patients' fellow eye. Fluorescein angiography revealed initial hypofluorescence without late leakage of the dye in all eyes included. Infrared images of scanning laser ophthalmoscopy revealed hyporeflectivity that corresponded to the ODP and the morning glory anomaly, while hyperreflectivity corresponded to the glaucomatous disc cupping. Mean ocular axial length was 23.63 ± 0.87 mm (range: 22.63–25.30 mm). Single-flash electroretinography results were normal in all affected eyes.

### 3.3. Preoperative OCT Findings

SD- and EDI-OCT clearly revealed the presence of excavation of the optic disc including ODP (Figures 1(b), 2(b), 5(b), and 6(c)) in all except one eye (case 4) and retinal schisis extending from the optic disc to the macula (Figures 1(b), 2(b), 3(c), 3(d), 5(b), and 6(c)). Macular detachment was observed in five patients (62.5%). An epipapillary membrane was observed at the cavitary sites of the disc in all eyes, regardless of the presence or absence of preoperative PVD (Figures 1(b), 2(b), 3(d), 5(b), and 6(c)). In 3 of 4 eyes without preoperative PVD (cases 1, 2, and 5), the posterior hyaloid membrane was directly attached on the epipapillary membrane, invaginating into the cavitary sites of the disc (Figures 1(b), 2(b), and 5(b)). In four eyes (cases 2, 3, 5, and 7), tunnel-like hyporeflectivities, which suggested a communication between the retinal schisis and the disc, were observed either beneath the ILM (two cases, Figures 2(b) and 6(c)) or at the outer nuclear layer (two cases, Figure 5(b)) at the vicinity of the disc. In case 4, funduscopy and SD-OCT failed to detect the excavation beneath the epipapillary membrane (Figures 3(b) and 3(d)).

### 3.4. Intraoperative Findings

During surgery, PVD was intentionally induced in four eyes. In two ODP maculopathy patients (cases 1 and 2), even when PVD was artificially induced, the hyaloid membrane was attached on the epipapillary membrane, which was firmly fixed into the ODP (Figures 1(c)–1(e)). The presence of epipapillary membrane could be strongly suspected by triamcinolone acetonide that was injected intraoperatively and attached on the optic disc in all patients. In all eyes, no retinal tear was observed around the disc. The epipapillary membrane that was present at the excavation sites of the disc was removed in all eyes. Intravitreal SF_6_ gas injection was administered to a patient with peripheral iatrogenic retinal breaks (case 7).

### 3.5. Postoperative Findings

**Figure 4:**
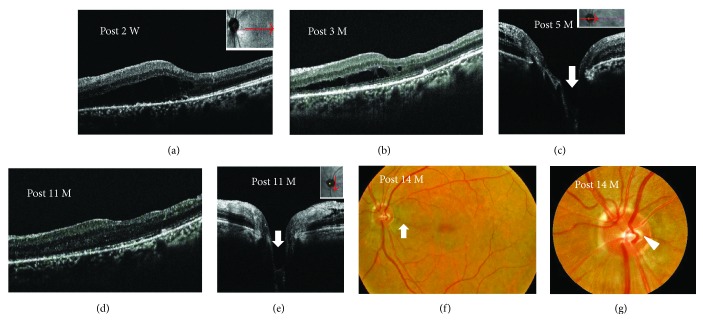
Findings after surgery in case 4. (a–e) Spectral domain optical coherence tomography images revealed that an optic disc coloboma (c, e, arrows) became clear after epipapillary membrane removal. Macular schisis also gradually decreased and was resolved 11 months after surgery (a, b, and d). (f, g) A retinal nerve fiber layer defect (f, arrow) corresponding to the coloboma (g, arrowhead) was evident together with the disappearance of the retinal schisis 14 months after surgery.

**Figure 5:**
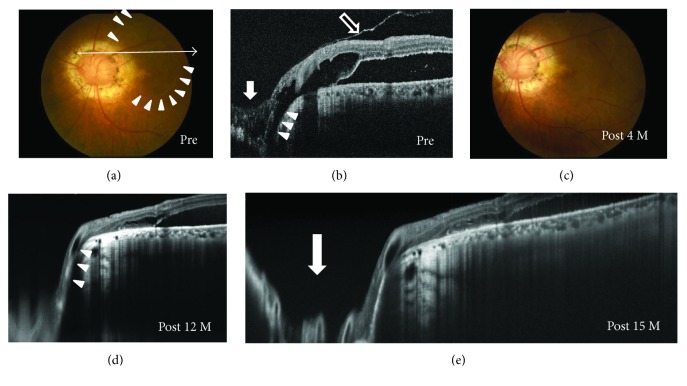
Photographs of the left eye in a 68-year-old patient with maculopathy associated with morning glory anomaly of the optic disc (case 5). (a, b) Preoperative images. Fundus photograph showed retinal detachment extending from the morning glory disc anomaly to the macula (a, arrowheads). Arrows indicate the optical coherence tomography (OCT) scan in Figure 5. A spectral domain OCT image showed retinal schisis with macular detachment and a tunnel-like hyporeflectivity extending from the retinal schisis toward the disc (b, arrowheads), suggesting a communication between the disc and retinal schisis (b). The hyaloid membrane (b, black arrow) was attached around the disc and on the epipapillary glial tissue (b, white arrow). (c) Macular detachment was reduced at 4 months after pars plana vitrectomy with epipapillary membrane removal. (d) Twelve months after surgery, an enhanced depth imaging OCT image revealed that the tunnel-like hyporeflectivity observed preoperatively was resolved with the reduction of the retinal schisis (arrowheads). (e) Fifteen months after surgery, retinal schisis was almost resolved and the optic disc cupping became deeper (arrow) than in pretreatment.

**Figure 6:**
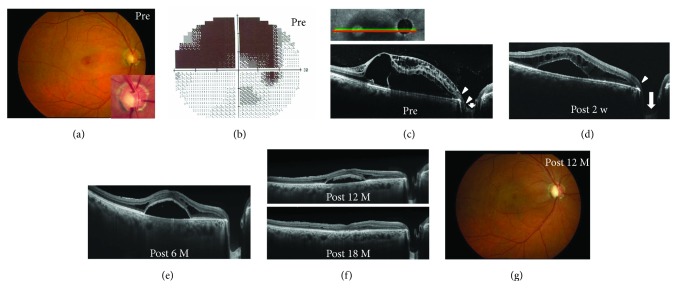
Photographs of the right eye in a 52-year-old patient with retinoschisis associated with glaucoma without posterior vitreous detachment (case 7). (a) Fundus photographs before surgery demonstrating retinal elevation, retinal folds extending from the macula to the optic disc, and glaucomatous optic disc cupping with nerve fiber layer defect but no obvious optic disc pit. (b) Humphrey threshold 30-2 perimetry before surgery showing decreased sensitivity at the Bjerrum area. (c–f) Horizontal scan images of enhanced depth imaging optical coherence tomography. An image before surgery showed retinal schisis with foveal detachment extending from the optic disc to the macula, the epipapillary membrane (c, arrow), and a shallow tunnel-like hyporeflectivity (c, arrowheads) directly connecting the retinal schisis and the 8 o' clock margin of the disc (c). The posterior hyaloid membrane was attached to the vicinty of the disc. Two weeks after pars plana vitrectomy, retinal schisis decreased with an obscure tunnel-like hyporeflectivity (d, arrowhead). The extent of the disc cupping was obviously deeper than preoperative one following the removal of the membrane tissue (d, arrow). Retinal schisis with macular detachment gradually decreased (d–f) and was completely resolved 18 months after surgery (f). (g) Fundus photograph showed the decrease of macular elevation at the macula but no optic disc pit at 12 months after surgery.

OCT revealed that the excavation of the optic disc became deeper than that of pretreatment after the surgery with the removal of the epipapillary membrane in all patients (Figures 1(g), 2(c), 4(c), 5(e), and 6(d)). The tunnel-like hyporeflectivities detected using OCT preoperatively became obscure from the early stage after PPV (Figures 2(c) and 6(d)). During follow-up, macular schisis gradually decreased and finally showed complete resolution in almost all patients (Figures 1(h), 2(d), 4(d), 5(e), and 6(g)). Macular detachment was also resolved in four patients and was reduced in one patient (case 5). In one patient (case 4), OCT revealed the optic disc coloboma that was preoperatively concealed beneath the epipapillary membrane (Figures 4(c) and 4(e)). One patient (case 2) developed a macular hole, which was closed following SF_6_ gas tamponade. None of the patients developed systemic or other ocular complications. Of the three patients with glaucoma, the mean deviation values on the perimetry remained unchanged compared with the preoperative values during follow-up in two patients (cases 7 and 8), while it remained unchanged until 19 months after PPV but deteriorated after 22 months in one patient (case 6), suggesting a progression of glaucoma but not perioperative mechanical damage on the disc.

### 3.6. Changes of CRT and BCVA

The mean CRT in the included patients was reduced significantly (*P* = 0.01 and 0.004, resp.) from 674.8 ± 235.0 *μ*m at pretreatment to 451.8 ± 199.5 *μ*m six months after surgery and to 290.9 ± 116.9 *μ*m at the final follow-up. BCVA at pretreatment was 0.5–0.9 in four eyes and 0.1–0.4 in the remaining four eyes. BCVA at the final follow-up was ≥1.0 in four eyes, 0.5–0.9 in three eyes, and 0.1–0.4 in one eye. Moreover, the mean logMAR value of the BCVA (0.12 ± 0.23) improved significantly compared with the pretreatment value (0.37 ± 0.31; *P* = 0.008).

### 3.7. Ophthalmic Pathology

**Figure 7:**
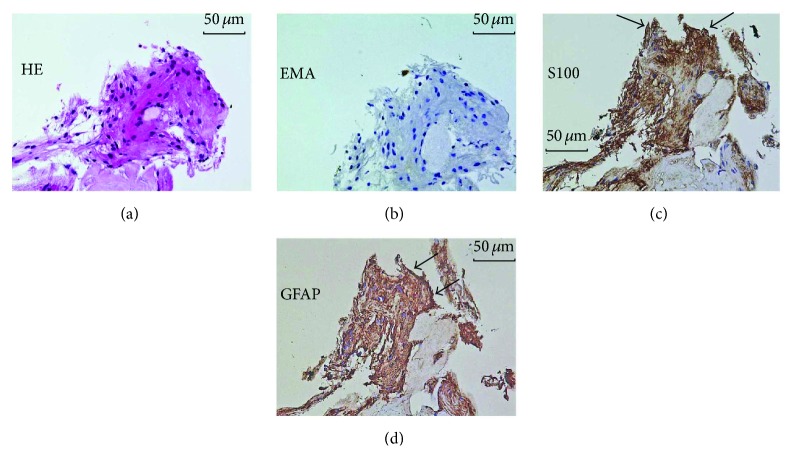
Histology (a) and immunohistochemistry (b–d) of a resected epipapillary membrane tissue in case 4. (a) Light micrograph demonstrating marked hyalinization. Spindle-shaped stromal cells were intermingled in the section (hematoxylin-eosin). (b) Immunochemistry for cytokeratin. Immunoreactivity was not observed. (c) Immunoreactivity for S100 was detected in the membrane (arrows). (d) Immunoreactivity for GFAP was strongly detected in the membrane (arrows).

**Figure 8:**
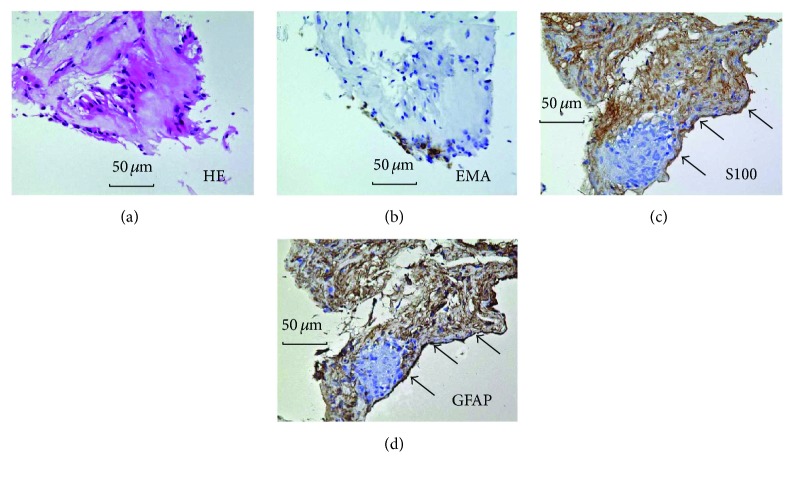
Histology (a) and immunohistochemistry (b–d) of a resected epipapillary membrane tissue in case 5. (a) Light micrograph showing marked hyalinization. Spindle-shaped stromal cells were intermingled in the section (hematoxylin-eosin). (b) Immunochemistry for cytokeratin. Immunoreactivity was not observed. (c) Immunoreactivity for S100 was strongly detected in the membrane (arrows). (d) Immunoreactivity for GFAP was strongly detected in the membrane (arrows).

The histology of the epipapillary membrane tissues resected in cases 4 and 5 revealed that spindle-shaped stromal cells were intermingled within the section (Figures 7(a) and 8(a)). Immunoreactivity for cytokeratin was not observed (Figures 7(b) and 8(b)). In contrast, immunoreactivity for S100 and GFAP was detected in the membrane (Figures 7(c), 7(d) and 8(c), 8(d)).

## 4. Discussion

In patients with maculopathy associated with congenital or acquired cavitary optic disc anomalies, we made the following observations: (1) an epipapillary membrane was observed in all patients via SD-OCT. (2) Maculoschisis was resolved with no recurrence regardless of the disease type or the presence or absence of preoperative PVD after PPV with epipapillary membrane removal in all patients. (3) The mean logMAR BCVA and CRT at the final follow-up significantly improved compared with pretreatment values. (4) Immunohistochemistry for GFAP and S100 proteins, which are markers for astrocytes, was positive in the resected membranes on the disc.

In all our patients without preoperative PVD, preoperative OCT revealed that the epipapillary membrane was present at the excavation sites of the disc, as shown in a recent study [17]. The hyaloid membrane was attached to not only the optic disc but also the epipapilary membrane in most of these patients. Moreover, we intraoperatively confirmed the connection between the epipapillary membrane and the hyaloid membrane, even though the latter was intentionally detached by PPV (Figures 1(c)–1(e)). The retinal schisis was resolved after PPV with the removal of both membranes, as shown in also a previously reported case [18]. In some patients with ODP maculopathy, actually, the retinal schisis did not improve or recur despite the PPV with only the removal of hyaloid membrane and/or ILM [9, 13, 14]. It is known that eyes with cavitary optic disc anomalies including ODP and glaucoma have congenital or acquired anatomical vulnerability at the optic disc in common [1, 2, 24]. Taken together, these results indicate that in some patients with cavitary optic disc anomaly-associated maculopathy without preoperative PVD, the epipapillary membrane, in concert with the hyaloid membrane, may play a role in the pathogenesis of the maculopathy by dragging these vulnerable disc tissues as the traction force to the disc. Postoperative resolution of the tunnel-like hyporeflectivity connecting the retinal schisis and the optic disc observed on preoperative OCT suggests a halt of the fluid flowing from the site with the disc anomalies to the retinal schisis.

It has been previously reported that ODP maculopathy patients with preoperative PVD are usually older, with no improvement of the retinal schisis and with deterioration of the BCVA postoperatively [15]. In this study, all patients with preoperative PVD had the epipapillary membrane on SD-OCT. Their retinal schisis and/or detachment was resolved after PPV with the removal of the epipapillary membrane. Furthermore, a tunnel-like hyporeflectivity connecting the schisis and the excavation site of the disc observed with SD-OCT preoperatively became obscured from the early stage after surgery [21]. Intrapapillary proliferation was observed in all of 16 ODP maculopathy eyes [17]. The proliferation that sequentially developed was likely to cause the development of cavity within the pit [17]. Therefore, our current results suggest that the growth of the residual epipapillary membrane continues drawing the vulnerable disc tissues, even after PVD spontaneously occurred, and is involved in the pathogenesis of this maculopathy with preoperative PVD.

In the present study, ILM removal was performed in all patients included to prevent the occurrence of epiretinal membrane after surgery. The procedure might affect surgical outcomes. However, a previous report showed that retinoschisis was resolved in almost all patients by performing vitrectomy with intensional generation of PVD but without ILM removal [9]. In a study with a large number of patients with ODP maculopathy, moreover, there was no association between ILM peeling and improvement of the macular schisis [14]. This may be because it is difficult to release the traction of hyaloid and/or epipapillary membranes from the disc just by performing ILM removal. Therefore, we speculate that ILM removal would be restrictive for improvement of retinoschisis in this disease complex.

Furthermore, we detected GFAP and S100 proteins in the resected epipapillary membrane, suggesting the existence of astrocytes. Astrocytes are glial cells surrounding vessels or nerve fibers. Differentiated perinatally, they subsequently proliferate in the optic nerve and spread from the retinal nerve fiber layer to the neural retina at the periphery via the optic disc [25]. In the normal retina, astrocytes exist around the retinal ganglion cell layer; however, they penetrate deeper into retinal layers upon retinal detachment [26]. From our surgical and immunohistochemical results, we speculated that astrocytes migrated into deeper retinal layers following the onset of retinal schisis and/or detachment and subsequently migrated to the epipapillary membrane via a communication between the retinal schisis and the optic disc. Our histological observations may support that the epipapillary membrane relates to the pathogenesis of some patients with optic disc anomaly-associated maculopathy, although further research is required to verify our speculation.

In case 4, optic disc coloboma [23], which was hidden by the epipapillary membrane preoperatively, was found after PPV with membrane removal. Therefore, this case may be called occult optic disc coloboma-associated maculopathy. Cases with retinal schisis extending from the disc to the macula despite having funduscopically normal optic disc have been recently reported [27]. These cases had a predisposition to occur in emmetropic eyes of older patients and in the presence of preoperative PVD. These features were consistent with case 4 in the present study. However, the presence of the membrane tissue on the disc has not been examined yet in these idiopathic cases. Therefore, further studies are needed to examine the presence of the epipapillary membrane in cases with idiopathic foveomacular retinal schisis.

This pilot study has some limitations. This is a retrospective study, with a small number of patients; prospective studies with a larger number are needed to examine the outcome of our surgical procedure. Moreover, the surgical procedure shown in the present study has a possibility of causing optic nerve damage in eyes undergoing surgery, because S100 and GFAP being positive on immunohistochemistry of the resected epipapillary membranes were markers of not only astrocytes but also neural tissues of the optic disc. We confirmed that none of the three eyes with glaucomatous cupping showed any exacerbation in the visual field defects in the early stages after the surgery. Histological results of the resected membranes demonstrated a variety of cellular components, in addition to being positive for GFAP and S100, which were not consistent with neural fibers in the optic disc. The results suggest that the cells in the isolated membranes are astrocytes rather than neural tissues. When the surgeon performed the surgical procedure, careful operation handling was required in order not to injure the optic nerve head. Additionally, damage of the disc should be routinely monitored by performing perimetry before and after surgery.

## 5. Conclusions

Epipapillary membrane was preoperatively observed in all patients with maculopathy associated with cavitary optic disc anomalies. After PPV with epipapillary membrane removal, the retinal schisis was completely or almost resolved regardless of the disease type or the presence or absence of preoperative PVD, and the mean CMT and BCVA significantly improved. Markers for astrocytes were positive in the immunohistochemistry of the resected membranes on the disc. Our current data suggest that epipapillary membrane is involved in the pathogenesis of some patients with maculopathy associated with cavitary optic disc anomalies as well as posterior hyaloid membrane. PPV with epipapillary membrane removal may be a useful treatment option for patients with this disease complex. In the future, multicentered studies comparing surgical outcomes of PPV with or without epipapillary membrane removal are needed to verify the present results.

## Figures and Tables

**Table 1:** Clinical characteristics of patients with maculopathy associated with cavitary optic disc anomalies.

Case	Age/sex	Follow-up period (months)	Cause of maculopathy	Macular schisis	Macular RD	Preoperative PVD	Epipapillary membrane	Decimal BCVA		Central macular thickness (*μ*m)			Others
								Pre	Final	Pre	Post 6 M	Final	Findings
1	20/M	23	Optic disc pit	+	+	−	+	0.7	1.2	747	309	251	
2	24/M	10	Optic disc pit	+	+	−	+	0.4	0.9	848	309	284	PPV → MH → SF_6_ gas injection → MH closed
3	82/F	27	Optic disc pit	+	+	+	+	0.1	0.2	662	324	128	Fellow eye: old morning glory syndrome
4	70/F	57	Optic disc coloboma	+	−	+	+	0.4	0.8	448	244	231	Fellow eye: optic disc coloboma without maculopathy
5	68/M	15	Morning glory disc anomaly	+	+	−	+	0.5	0.8	585	623	460	
6	79/M	27	Glaucoma	+	−	+	+	0.6	1.0	444	374	326	VF defect remained unchanged postoperatively until 19 months
7	52/M	27	Glaucoma	+	+	−	+	0.3	1.0	1055	672	243	VF defect remained unchanged postoperatively
8	77/M	18	Glaucoma	+	−	+	+	0.8	1.0	771	759	404	VF defect remained unchanged postoperatively

RD: retinal detachment; PVD: posterior vitreous detachment; BCVA: best-corrected visual acuity; PPV: pars plana vitrectomy; MH: macular hole; VF: visual field.
